# The impact of body roundness index (BRI) on obstructive sleep apnea (OSA) severity: an exploratory sequential mixed-methods study

**DOI:** 10.3389/fmed.2026.1885036

**Published:** 2026-07-29

**Authors:** Yingchun Zhang, Heyan Ouyang, Zhenzhen Zheng, Huan Li, Shuyue Zhou, Renkang Zhong, Jingxuan Lei, Chengsong Liao, Junfen Cheng, Min Peng, Xuliang Chen, Baozhi Zhang, Riken Chen

**Affiliations:** The Second Affiliated Hospital of Guangdong Medical University, Zhanjiang, Guangdong, China

**Keywords:** body roundness index, disease severity, gender differences, mixed-methods research, obstructive sleep apnea, patient perspective

## Abstract

**Background:**

Screening for obstructive sleep apnea (OSA) in primary care is hindered by the lack of readily available, pathophysiologically informed tools. The Body Roundness Index (BRI), a novel metric reflecting central obesity, has unclear clinical applicability and efficacy boundaries, particularly in explaining the paradox of “high BRI without severe OSA.”

**Methods:**

We employed an explanatory sequential mixed-methods design. In Phase I, ordered logistic regression and ROC analysis quantified the association and diagnostic value of BRI with OSA severity in 2,596 suspected OSA patients. In Phase II, semi-structured interviews and thematic analysis explored disease attribution and behavioral coping in 25 patients categorized into a “Contradictory Group” (high BRI, non-severe OSA; *n* = 8) and a “Typical Group” (high BRI, severe OSA; *n* = 17).

**Results:**

BRI was an independent predictor of OSA severity (OR = 1.238, 95% CI: 1.159–1.322, *P* < 0.001). Its diagnostic performance for severe OSA (AUC = 0.668) was comparable to BMI (AUC = 0.674) but inferior to neck circumference (AUC = 0.711). Efficacy was higher in males (AUC = 0.645) but limited for mild-to-moderate OSA (AUC = 0.532 and 0.490 for mild and moderate groups, respectively). Qualitatively, the Contradictory Group systematically attributed symptoms to localized structural issues, complex comorbidities, or physiological events rather than systemic obesity, forming a disease perception model distinct from the typical “obesity-obstruction” paradigm.

**Conclusion:**

While BRI provides a pathophysiologically grounded alternative to BMI for severe OSA risk assessment, combining BRI, BMI, and neck circumference offers modest incremental diagnostic value. We advocate shifting OSA screening toward a stepwise, patient-centered pathway that prioritizes neck circumference and systematically integrates multidimensional clinical characteristics and health beliefs.

## 1 Introduction

Obstructive sleep apnea (OSA) is a common disorder characterized by recurrent upper airway collapse during sleep, leading to intermittent hypoxia and fragmented sleep. It represents an independent risk factor for hypertension, cardiovascular and cerebrovascular diseases, and cognitive impairment, constituting a significant public health burden (1, 2). Although polysomnography (PSG) serves as the gold standard for diagnosing OSA, its operational complexity and high cost limit its application in population screening and primary care settings (3). Consequently, identifying accessible, objective screening markers grounded in pathophysiological mechanisms is crucial for achieving early OSA recognition.

Central obesity represents the most fundamental modifiable risk factor for OSA (4, 5), with mechanical compression from peripharyngeal fat deposits serving as a well-established pathophysiological mechanism (6, 7). However, commonly used clinical obesity metrics each have limitations: Body Mass Index (BMI) fails to distinguish fat from muscle; waist circumference struggles to accurately reflect three-dimensional fat distribution; and neck circumference measurements are susceptible to operator variability (8, 9). The Body Roundness Index (BRI) is a novel anthropometric indicator proposed in recent years. By integrating height and waist circumference, it theoretically quantifies central obesity more accurately (10, 11) and has been demonstrated to correlate closely with cardiovascular metabolic risk (12–14). Recent evidence further indicates that novel anthropometric indices, including BRI, are not only associated with sleep-related respiratory parameters but also serve as significant predictors of elevated cardiovascular risk specifically in patients with obstructive sleep apnea syndrome (15). However, translating BRI into a reliable OSA screening tool faces a significant clinical paradox: if BRI accurately reflects core pathology, why do numerous” high BRI but non-severe OSA” individuals exist in practice?

Most existing studies have stopped at verifying the statistical association between BRI and OSA, without providing a satisfactory explanation for this paradox (16, 17). This leaves two critical questions unresolved: First, where exactly are the diagnostic efficacy boundaries of BRI? (For example, for which genders or severity levels of OSA is it most effective?) Second, what are the fundamental causes of these performance boundaries? (e.g., which competing etiologies or patient factors interfere?) This “black box” state severely hinders the integration of BRI into effective clinical pathways.

To resolve this paradox, OSA must be reinterpreted as a disease interwoven with bio-psycho-social models. Patients’ symptom experiences, disease attribution, and health beliefs are not merely mediators influencing healthcare-seeking behavior; they themselves may signify distinct etiological subtypes or “phenotypes” (18, 19). Thus, singular quantitative association studies are insufficient to guide precision practice. Mixed-methods sequential design offers an ideal framework: its quantitative phase objectively maps indicator “performance profiles,” while the qualitative phase delves into the human dimensions underlying the data, seeking mechanistic explanations for “why.”

Therefore, this study aims to transcend simple validation of BRI’s diagnostic efficacy. We employ an explanatory sequential mixed-methods design to achieve three integrated objectives: (1) Systematic Mapping: Comprehensively evaluate BRI’s diagnostic efficacy, identifying its ADVANTAGEOUS populations (e.g., men with severe OSA) and inherent limitations. (2) In-depth exploration: Through patient narratives, uncover unique disease cognition models and behavioral logic underlying “contradictory” phenomena. (3) Integrative analysis: Merge objective and subjective evidence to establish a preliminary pathway for feasible, individualized OSA screening and management grounded in both objective metrics and patient narratives, providing dual scientific and humanistic foundations.

## 2 Materials and methods

### 2.1 Study design

This study employed an explanatory sequential mixed-methods design comprising two consecutive phases. Phase I was a cross-sectional quantitative study assessing the association and diagnostic efficacy between the Body Roundness Index (BRI) and OSA severity. Phase II was a descriptive qualitative study exploring patients’ disease cognition, symptom experiences, and coping strategies, specifically targeting the paradoxical phenomenon of “high BRI without severe OSA” identified in Phase I to explore potential protective factors or diagnostic limitations. The two phases were integrated using a “linking approach,” where the qualitative sampling framework and interview guide were directly derived from the quantitative results. This study adheres to STROBE guidelines for observational studies (20), COREQ guidelines for qualitative research (21, 22), and standards for reporting mixed-methods research (23, 24).

### 2.2 Study population

#### 2.2.1 Quantitative phase

We retrospectively analyzed patients referred for suspected OSA evaluated at the Sleep Medicine Center, Second Affiliated Hospital of Guangdong Medical University, from April 2024 to April 2025. Inclusion criteria were age ≥ 18 years, completion of overnight polysomnography (PSG), complete clinical records, and voluntary participation with written informed consent. Patients were excluded if they had central or mixed sleep apnea; severe hepatic/renal insufficiency, malignant tumors, or neurological disorders affecting respiration; psychiatric disorders; were pregnant or lactating; or had > 10% missing data.

Based on the apnea-hypopnea index (AHI) measured by PSG (19), participants were categorized into Normal (AHI < 5 events/h), Mild (5 ≤ AHI < 15), Moderate (15 ≤ AHI < 30), and Severe (AHI ≥ 30) groups. Based on previous epidemiological data (25, 26), the minimum required sample size was calculated as 622 (detailed parameters and formula are provided in Supplementary material S1). Ultimately, 2,596 participants were enrolled, providing ample statistical power for multivariate and subgroup analyses.

The primary outcome was OSA severity based on AHI. The core exposure variable, BRI, was calculated using standard soft tape measurements of height and waist circumference (in centimeters, converted to meters for calculation) according to established formulas (10) (see Supplementary material S2). Covariates included demographics, BMI classification, neck circumference, clinical history (e.g., hypertension, diabetes), OSA risk assessment, and Epworth Sleepiness Scale (ESS) scores.

#### 2.2.2 Qualitative phase

Using purposive sampling based on Phase I results, we recruited 25 participants. To explore the clinical paradox of high visceral adiposity without severe OSA, participants were categorized into a “Contradiction Group” (*n* = 8; BRI ≥ 5.6 and AHI < 30 events/h) and a “Typical Group” (*n* = 17; BRI ≥ 5.6 and AHI ≥ 30 events/h). The BRI cutoff of 5.6 represented the upper quartile of our study population. Groups were balanced for gender, age, and comorbidities. Inclusion required normal cognitive/language abilities and written informed consent. Exclusions included communication barriers, psychiatric disorders, refusal of audio recording, or recent (within 1 month) OSA-related interventions.

Sample size was determined by the principle of “information saturation” (27), which was achieved after interviewing the 25th participant. Semi-structured interviews were conducted using a guide developed from our quantitative findings and relevant literature (see Supplementary material S3). Core dimensions explored included disease attribution, symptom experience, behavioral coping strategies, and perceptions of body shape and OSA.

### 2.3 Data collection

#### 2.3.1 Quantitative phase

General demographic data were collected via structured questionnaires. Anthropometric measurements were performed in triplicate by trained personnel following standardized procedures, with mean values recorded. All participants underwent overnight polysomnography (PSG, ≥ 7 h) (19), with results independently scored by two sleep physicians; discrepancies were resolved through consensus. OSA-related symptoms and clinical risks were assessed using the Epworth Sleepiness Scale (ESS) (28) and the Berlin Questionnaire (29), with participants self-reporting snoring frequency and breathing arousals.

#### 2.3.2 Qualitative phase

Two trained researchers conducted semi-structured interviews between July and September 2025. Each session lasted 30–60 min in a quiet, private setting or via secure video conferencing. Interviews were audio-recorded with concurrent field notes documenting nonverbal behaviors. Researchers compiled comprehensive memos within 24 h, cross-referencing recordings and notes to clarify ambiguous information and ensure data fidelity.

### 2.4 Ethical considerations

This study was approved by the Ethics Committee of the Second Affiliated Hospital of Guangdong Medical University (Approval No: PJKT2025-050). All participants provided written informed consent. Data were anonymized using identifier codes; qualitative recordings and transcripts were stored on an encrypted server accessible only to authorized personnel. Psychological support resources were available to qualitative participants as needed.

### 2.5 Data analysis

#### 2.5.1 Quantitative analysis

Statistical analyses were performed using R 4.5.1. Continuous variables were expressed as mean ± standard deviation or median [interquartile range] based on normality, with group comparisons via one-way ANOVA or Kruskal-Wallis H tests. Categorical variables were presented as frequencies (percentages) and compared using chi-square tests. Correlations between BRI and OSA severity were assessed using Pearson or Spearman tests.

Ordered multivariate logistic regression was performed to determine whether BRI is an independent risk factor for OSA severity, adjusting for age, gender, BMI, neck circumference, and metabolic comorbidities. To ensure the validity of our multivariable models containing overlapping anthropometric metrics, collinearity diagnostics were formally performed. Variables with a Variance Inflation Factor (VIF) < 5 were considered acceptable, indicating that multicollinearity did not critically bias the regression estimates. Stratified analyses and interaction terms evaluated moderating effects of BMI. Diagnostic performance of BRI alone and combined with BMI and neck circumference was assessed using ROC curve analysis, reporting AUC, sensitivity, specificity, and optimal cutoffs. DeLong’s test compared AUC differences between models. Two-tailed *P* < 0.05 was considered statistically significant.

#### 2.5.2 Qualitative analysis

Qualitative data were managed using NVivo 15.0 and analyzed using thematic analysis informed by grounded theory coding principles. The process involved: (1) familiarization with transcripts; (2) open coding to extract initial concepts; (3) axial coding to form subthemes; and (4) selective coding to refine core themes. Three researchers conducted independent coding; discrepancies were resolved through consensus. Rigor was ensured via researcher triangulation, iterative refinement, and member checking.

#### 2.5.3 Data integration

A visual joint display synthesized quantitative and qualitative findings. Quantitative results (e.g., statistical associations, subgroup disparities) were juxtaposed with qualitative themes (e.g., alternative disease interpretations in the contradiction group, typical cognitive characteristics) to cross-validate findings and resolve discrepancies. This integration informed a multilevel moderation framework elucidating the interplay among individual health cognition, clinical characteristics, and behavioral patterns.

## 3 Results

### 3.1 Quantitative findings

#### 3.1.1 Baseline characteristics

Of 2,596 enrolled participants, OSA severity was categorized as normal (28.9%), mild (22.4%), moderate (18.3%), and severe (30.4%). The mean age was 49.8 ± 15.8 years, 76.6% were male, and mean BMI was 26.2 ± 1.51 kg/m^2^. Increasing OSA severity was significantly associated with higher BMI, larger neck circumference, and lower minimum oxygen saturation (all *P* < 0.001). The severe OSA group also showed higher proportions of male sex, obesity, high-risk OSA screening scores, and severe daytime sleepiness (Table 1).

**TABLE 1:** Baseline characteristics of study subjects and intergroup comparisons.

Variable	OSA severity					*P*-value
	Normal (*n* = 751)	Mild (*n* = 581)	Moderate (*n* = 474)	Severe (*n* = 790)	Test statistic	
BRI	4.10 [3.20, 5.10]	4.50[3.70, 5.30]	4.60[3.60, 5.40]	5.20[4.30, 6.30]	*H* = 222.46	< 0.001
Age	48.50 ± 15.97	53.37 ± 16.02	55.68 ± 16.61	47.85 ± 15.24	*F* = 34.25	< 0.001
Minimum blood oxygen saturation	90 [86.6, 92.0]	84 [80.0, 87.5]	80 [75.0, 83.07]	69 [58.0, 77.0]	*H* = 1400.12	< 0.001
Neck circumference	36.25 ± 3.86	37.42 ± 3.79	38.01 ± 3.72	39.97 ± 3.89	*F* = 126.73	< 0.001
Waist circumference	88.58 ± 11.38	91.67 ± 11.52	92.70 ± 12.05	99.41 ± 11.66	*F* = 118.32	< 0.001
BMI classification					χ^2^ = 267.20	< 0.001
Underweight	64 (8.5%)	28 (4.8%)	32 (6.8%)	12 (1.5%)		
Normal	293 (39.0%)	175 (30.1%)	134 (28.3%)	126 (15.9%)		
Obese	136 (18.1%)	120 (20.7%)	119 (25.1%)	377 (47.7%)		
Overweight	258 (34.4%)	258 (44.4%)	189 (39.9%)	275 (34.8%)		
Gender					χ^2^ = 139.18	< 0.001
Male	492 (65.5%)	418 (71.9%)	366 (77.2%)	712 (90.1%)		
Female	259 (34.5%)	163 (28.1%)	108 (22.8%)	78 (9.9%)		
OSASH risk assessment					χ^2^ = 243.80	< 0.001
Low risk	558 (74.3%)	319 (54.9%)	265 (55.9%)	274 (34.7%)		
High risk	193 (25.7%)	259 (44.5%)	208 (43.9%)	515 (65.2%)		
Daytime sleepiness level					χ^2^ = 197.3	< 0.001
Normal	595 (79.2%)	420 (72.3%)	354 (74.7%)	408 (51.6%)		
Mild	73 (9.7%)	85 (14.6%)	71 (15.0%)	141 (17.8%)		
Moderate	47 (6.3%)	47 (8.1%)	24 (5.1%)	100 (12.7%)		
Severe	36 (4.8%)	29 (5.0%)	25 (5.3%)	141 (17.8%)		
Smoking status					χ^2^ = 82.98	< 0.001
Smoking	214 (28.5%)	196 (33.7%)	151 (31.9%)	357 (45.2%)		
Occasionally	365 (48.6%)	214 (36.8%)	166 (35.0%)	274 (34.7%)		
Non-smoker	172 (22.9%)	171 (29.4%)	157 (33.1%)	159 (20.1%)		
Drinking habits					χ^2^ = 57.76	< 0.001
Drinking	132 (17.6%)	134 (23.1%)	112 (23.6%)	228 (28.9%)		
Occasionally	427 (56.9%)	266 (45.8%)	195 (41.1%)	381 (48.2%)		
Do not drink alcohol	192 (25.6%)	181 (31.2%)	167 (35.2%)	181 (22.9%)		
History of hypertension					χ^2^ = 50.83	< 0.001
Yes	137 (18.2%)	174 (29.9%)	154 (32.5%)	260 (32.9%)		
None	614 (81.8%)	407 (70.1%)	320 (67.5%)	530 (67.1%)		
Diabetes history					χ^2^ = 7.83	0.05
Yes	46 (6.1%)	46 (7.9%)	48 (10.1%)	73 (9.2%)		
None	705 (93.9%)	535 (92.1%)	426 (89.9%)	717 (90.8%)		
History of coronary heart disease					χ^2^ = 3.88	0.274
Yes	35 (4.7%)	29 (5.0%)	32 (6.8%)	51 (6.5%)		
None	716 (95.3%)	552 (95.0%)	442 (93.2%)	739 (93.5%)		
History of cerebrovascular disease					χ^2^ = 2.94	0.401
Yes	24 (3.2%)	24 (4.1%)	23 (4.9%)	26 (3.3%)		
None	727 (96.8%)	557 (95.9%)	451 (95.1%)	764 (96.7%)		
History of rhinitis					χ^2^ = 74.51	< 0.001
Yes	166 (22.1%)	120 (20.7%)	89 (18.8%)	166 (21.0%)		
None	85 (11.3%)	119 (20.5%)	120 (25.3%)	82 (10.4%)		
Not sure	500 (66.6%)	342 (58.9%)	265 (55.9%)	542 (68.6%)		
History of pharyngitis					χ^2^ = 70.32	< 0.001
Yes	96 (12.8%)	67 (11.5%)	58 (12.2%)	92 (11.6%)		
None	85 (11.3%)	121 (20.8%)	121 (25.5%)	88 (11.1%)		
Not sure	570 (75.9%)	393 (67.6%)	295 (62.2%)	610 (77.2%)		
Insomnia status					χ^2^ = 88.87	< 0.001
Yes	47 (6.3%)	27 (4.6%)	17 (3.6%)	22 (2.8%)		
None	81 (10.8%)	121 (20.8%)	123 (25.9%)	85 (10.8%)		
Occasionally	623 (83.0%)	433 (74.5%)	334 (70.5%)	683 (86.5%)		

BRI, Body Roundness Index; BMI, Body Mass Index; OSA, Obstructive Sleep Apnea; OSASH, Obstructive Sleep Apnea Severity Classification; ESS, Epworth Sleepiness Scale. Data are presented as mean ± standard deviation, median [interquartile range], or frequency (percentage) as appropriate. F statistic is derived from one-way ANOVA; H statistic is derived from Kruskal-Wallis H test; χ^2^ statistic is derived from Pearson’s chi-square test. *P* < 0.05 was considered statistically significant.

#### 3.1.2 Association between BRI and OSA severity

**FIGURE 1:**
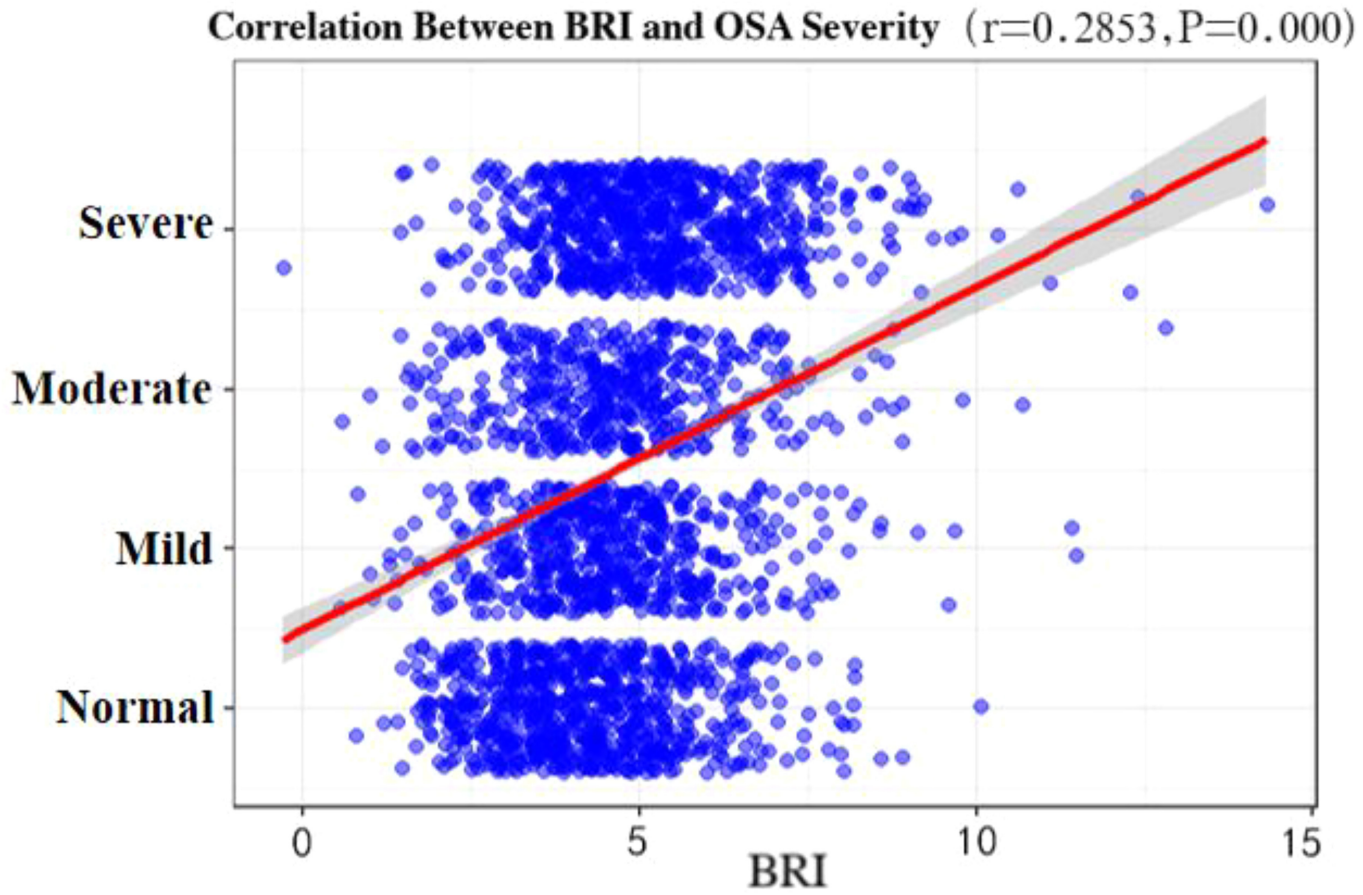
Scatterplot of BRI and OSA severity. The scatterplot illustrates the positive correlation between the Body Roundness Index (BRI) and the Apnea-Hypopnea Index (AHI)-based OSA severity groups. The red solid line represents the linear fit, and the gray shaded area denotes the 95% confidence interval. R represents the correlation coefficient (*r* = 0.2853, *P* = 0.000).

BRI demonstrated a significant positive correlation with OSA severity (*r* = 0.285, *P* < 0.001) (Figure 1). This association was weaker than that of waist circumference (*r* = 0.346) and neck circumference (*r* = 0.369), but stronger than age (*r* = −0.0168). Minimum oxygen saturation showed the strongest correlation (*r* = −0.734).

Ordered multivariate logistic regression revealed that, after adjusting for age, sex, BMI, and minimum SpO2, each unit increase in BRI was associated with a 23.8% higher risk of increased OSA severity (OR = 1.238, 95% CI: 1.159–1.322, *P* < 0.001). Male sex, obesity, and severe daytime sleepiness were also independent risk factors (Table 2). Collinearity diagnostics indicated acceptable levels among all independent variables (VIF range: 1.07–4.27; all VIF < 5), supporting the stability of the independent association observed for BRI.

**TABLE 2:** Correlation between BRI and OSA severity and multivariate regression analysis table.

Variable	Correlation analysis		Multivariate analysis		
	Correlation Coefficient (r/ρ)	*P*-value	Coefficient (Standard Error)	OR (95% CI)	*P*-value
BRI	0.2853	0.000	0.2135 (0.0337)	1.2381 (1.1588–1.3227)	< 0.001
Age	−0.0168	0.3913	0.011 (0.0026)	1.011 (1.0059–1.0162)	< 0.001
Minimum blood oxygen saturation	−0.7345	< 0.001	−0.1763 (0.006)	0.8384 (0.8286–0.8482)	< 0.001
Neck circumference	0.3689	< 0.001	0.0596 (0.0149)	1.0614 (1.0308–1.0929)	< 0.001
Waist circumference	0.3465	< 0.001	0.0496 (0.0112)	1.0508 (1.028–1.0742)	< 0.001
Gender (Male vs. Female)	−0.2278	< 0.001	0.9233 (0.0957)	2.5175 (2.0871–3.0366)	< 0.001
BMI classification	−0.121	< 0.001			
Underweight (vs. Normal)			−0.2594 (0.1842)	0.7715 (0.5377–1.1069)	0.159
Overweight (vs. Normal)			0.366 (0.0988)	1.4419 (1.1881–1.7499)	0.002
Obesity (vs. Normal)			0.7531 (0.128)	2.1235 (1.6523–2.7291)	< 0.001
OSASH risk assessment	0.3855	< 0.001			
High risk (vs. low risk)			0.563 (0.0981)	1.756 (1.449–2.1281)	< 0.01
Daytime sleepiness level	0.0812	< 0.001			
Mild (vs. Normal)			0.427 (0.1163)	1.5327 (1.2202–1.9253)	< 0.001
Moderate (vs. Normal)			0.6139 (0.1478)	1.8475 (1.3829–2.4683)	< 0.001
Severe (vs. Normal)			1.1068 (0.1538)	3.0247 (2.2375–4.0889)	< 0.001
Smoking status	0.0954	< 0.001			
Occasional (vs. Regular)			−0.203 (0.1682)	0.8163 (0.587–1.1351)	0.228
Smoking (vs. normal)			−0.0302 (0.1417)	0.9702 (0.7349–1.281)	0.831
Drinking status	−0.0396	0.0436			
Drinking (vs. normal)			−0.0757 (0.1426)	0.9271 (0.701–1.2261)	0.596
Occasional (vs. Normal)			−0.1581 (0.1578)	0.8537 (0.6267–1.1631)	0.316
History of hypertension (present vs. absent)	0.1253	< 0.001	0.0301 (0.0964)	1.0306 (0.8532–1.2449)	0.754
Diabetes (present vs. absent)	0.048	0.0146	0.0316 (0.1373)	1.0321 (0.7886–1.3509)	0.818
History of coronary heart disease (present vs. absent)	0.0346	0.0781	0.2195 (0.1676)	1.2454 (0.8968–1.7296)	0.190
History of cerebrovascular disease (present vs. absent)	0.004	0.8395	−0.0764 (0.2012)	0.9264 (0.6246–1.3742)	0.704
History of rhinitis	−0.0144	0.4638			
Present (vs. Absent)			0.136 (0.5595)	1.1456 (0.3826–3.4301)	0.808
Unclear (vs. absent)			0.1539 (0.563)	1.1664 (0.3869–3.5166)	0.785
History of pharyngitis	−0.0066	0.7387			
Present (vs. Absent)			−1.3252 (0.6329)	0.2657 (0.0769–0.9187)	0.036
Unclear (vs. None)			−1.1308 (0.6362)	0.3228 (0.0928–1.1231)	0.076
Insomnia	−0.0341	0.0827			
Present (vs. Absent)			−0.3291 (0.3928)	0.7196 (0.3332–1.554)	0.402
Occasionally (vs. None)			0.1866 (0.3979)	1.2051 (0.5525–2.6286)	0.639

Coefficient (Standard Error) and OR (95% CI) are calculated using ordered multivariate logistic regression, adjusting for age, gender, BMI, neck circumference, waist circumference, and metabolic comorbidities. Multi-collinearity was formally tested, with all Variance Inflation Factors (VIF) < 3.2. Two-tailed *P* < 0.05 indicates statistical significance.

#### 3.1.3 Subgroup and interaction analyses

**FIGURE 2:**
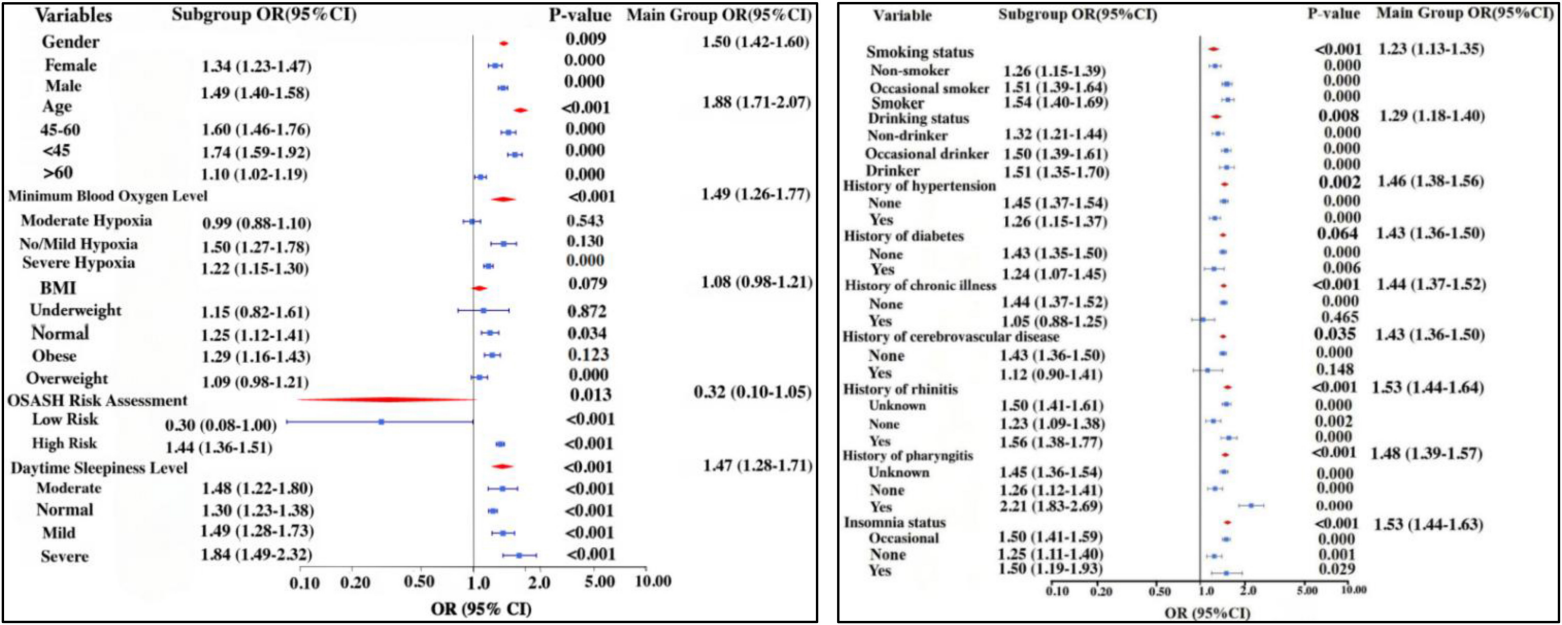
Forest plot and interactions for subgroup analysis of BRI correlation with OSA severity. Odds ratios (OR) and 95% confidence intervals (CI) are illustrated. The blue square represents the subgroup OR, and the horizontal lines represent the 95% CI. Minimum blood oxygen saturation classifications are defined as: Mild hypoxia: > 90%; Moderate hypoxia: 85%–90%; Severe hypoxia: < 85%. Interaction tests were performed to assess effect modification across subgroups.

**FIGURE 3:**
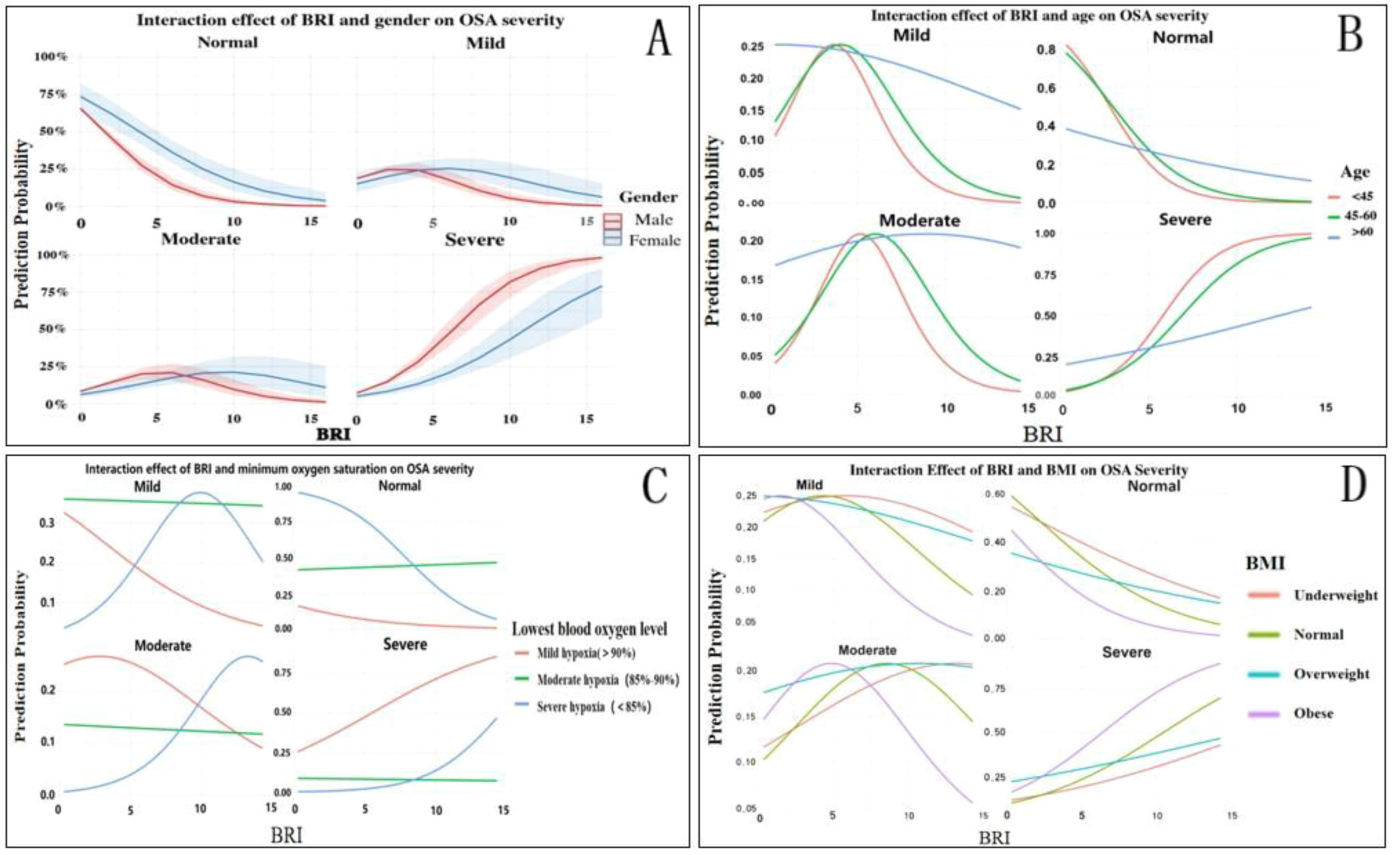
Interaction effect diagram of key modifying factors (gender, age, minimum blood oxygen level, and BMI) with BRI. **(A)** BRI and gender: Predicted probability of OSA severity across BRI values in males and females. **(B)** BRI and age: Association between BRI and OSA severity probability across age groups (<45, 45–60, >60 years). **(C)** BRI and minimum blood oxygen level: BRI-related OSA severity probability stratified by hypoxemia severity (mild: >90%; moderate: 85–90%; severe: < 85%). **(D)** BRI and BMI: Effect of BRI on OSA severity probability across BMI categories (Underweight, Normal, Overweight, Obese). The shaded bands surrounding the prediction curves represent the 95% confidence intervals.

Subgroup analyses revealed significant effect modification. The BRI-OSA association was stronger in males (OR = 1.49, 95% CI: 1.40–1.58), individuals aged < 45 years (OR = 1.74, 95% CI: 1.59–1.92), and those with pharyngitis history (OR = 2.21, P < 0.001) (Figure 2). Interaction tests confirmed that sex, age, hypoxia severity, daytime sleepiness, and pharyngitis history significantly moderated this association (all *P* < 0.05) (Figure 3).

#### 3.1.4 Diagnostic performance

**FIGURE 4:**
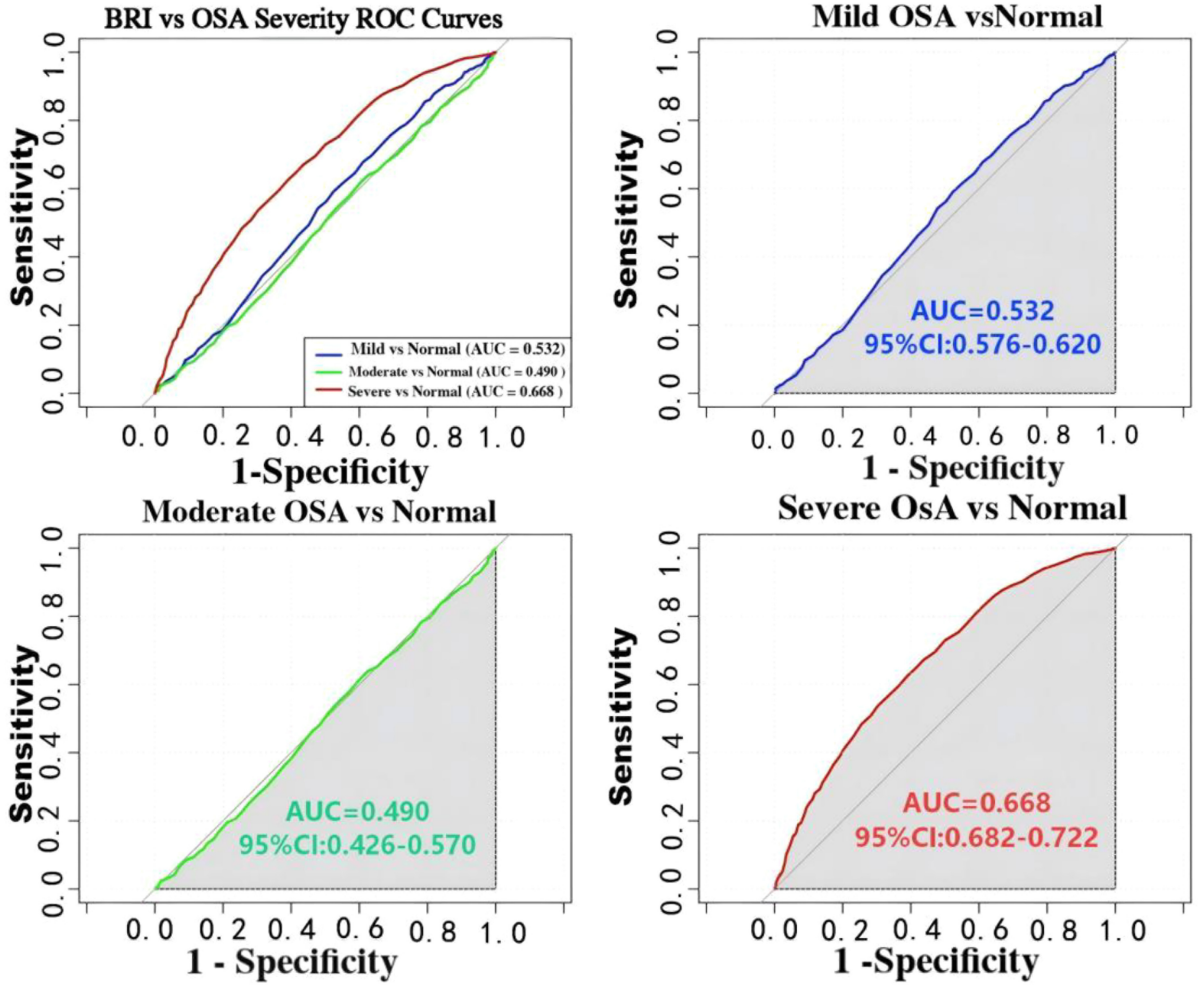
Predictive performance of BRI for different severity levels of OSA. ROC curves show the diagnostic performance of the Body Roundness Index (BRI) in predicting Mild OSA vs. Normal, Moderate OSA vs. Normal, and Severe OSA vs. Normal. AUC, Area Under the ROC Curve; CI, Confidence Interval. Colored lines represent different severity comparison models.

**FIGURE 5:**
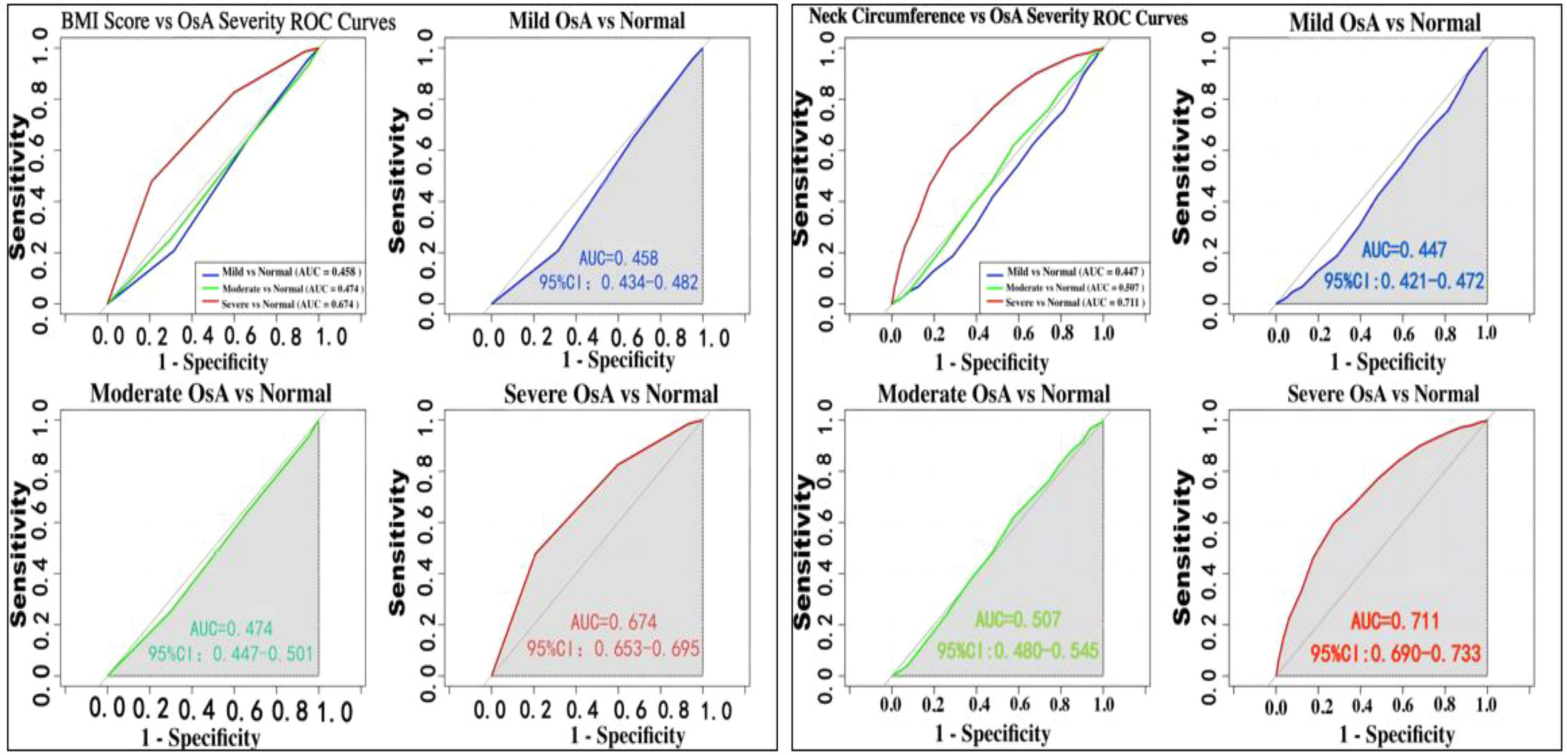
Predictive performance of BMI and Neck circumference for different OSA severity levels. Comparative ROC analysis of Body Mass Index (BMI, left panels) and Neck Circumference (right panels) across different severity thresholds. AUC, Area Under the Curve; CI, Confidence Interval.

**FIGURE 6:**
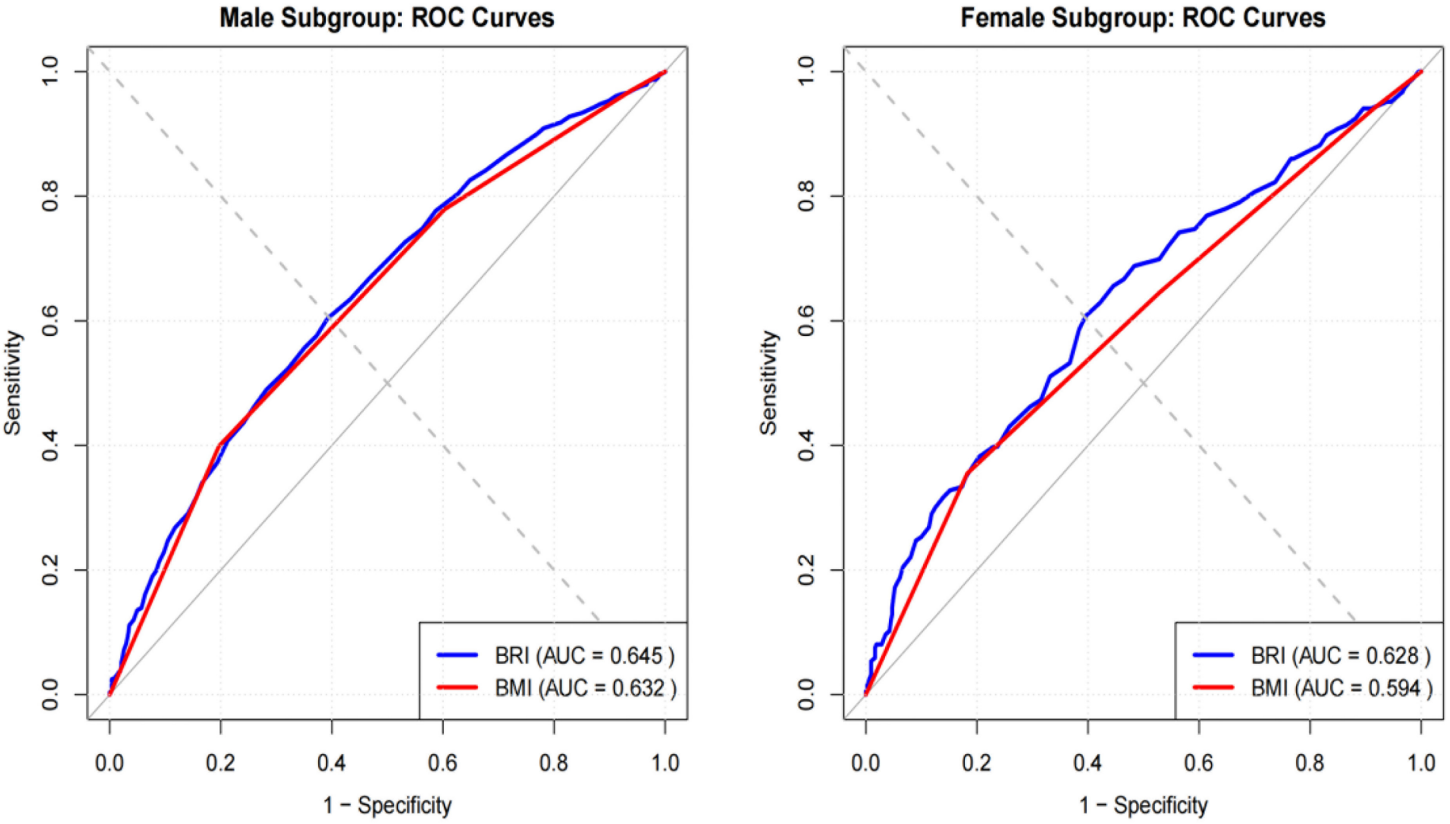
Predictive performance of BRI and BMI for severe OSA in gender subgroups. Gender-stratified ROC curves comparing the diagnostic efficacy of BRI (blue line) and BMI (red line) for severe OSA in males (left panel) and females (right panel).

**FIGURE 7:**
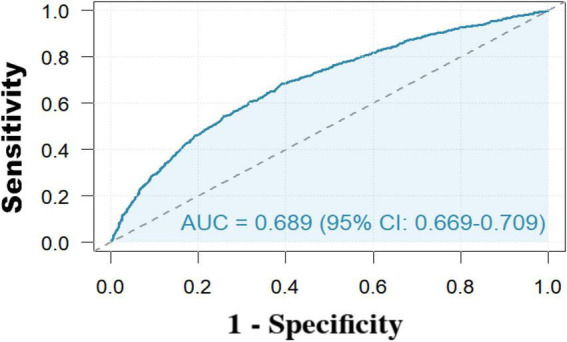
Predictive performance of the combined model (BRI+BMI+neck circumference) for severe OSA. ROC curve illustrating the diagnostic performance of the integrated model combining Body Roundness Index (BRI), Body Mass Index (BMI), and neck circumference.

For distinguishing severe OSA, BRI yielded an AUC of 0.668 (95% CI: 0.646–0.690), with an optimal cutoff of 4.75 (sensitivity 62.5%, specificity 61.0%). Its performance was comparable to BMI (AUC = 0.674) but inferior to neck circumference (AUC = 0.711). Sex-stratified analysis showed higher diagnostic efficacy in males (AUC = 0.645) than females (AUC = 0.628). A combined model (BRI + BMI + neck circumference) yielded an AUC of 0.689, which did not exceed that of neck circumference alone (AUC = 0.711) and suggested information redundancy among collinear obesity indicators (Figures 4–7; Supplementary Table S1).

### 3.2 Qualitative findings

The qualitative phase included 25 participants: 8 in the “Contradictory Group” (BRI ≥ 5.6, AHI < 30 events/h) and 17 in the “Typical Group” (BRI ≥ 5.6, AHI ≥ 30 events/h) (Supplementary Table S2). Mean age was 43.96 ± 11.23 years, and 48.0% were male.

#### 3.2.1 Disease attribution in the contradictory group

Thematic analysis revealed divergent cognitive models from the classic “obesity-obstruction” paradigm. Participants systematically shifted attribution toward localized or alternative explanations, categorized into five narrative types: (1) localized symptoms (e.g., excessive phlegm); (2) upper airway structural abnormalities; (3) complex comorbidity dominance (e.g., heart failure); (4) physiological life events (e.g., postpartum changes); and (5) cyclical process perceptions (Table 3).

**TABLE 3:** Analysis of disease narrative types in the conflict group.

Narrative type	Core attribution	Patient	Key thematic node (reference point) for this patient	Representative narrative of this patient
1. Local Symptom Attribution Type	Attributing OSA to a prominent local symptom.	P-C-02	1.1 Excessive phlegm as core symptom and attribution (1)	“I have phlegm. As soon as I lie down, it comes up. It must be related, right? Isn’t it because there’s phlegm in the throat, causing breathing difficulties that lead to snoring? I think it affects things to some extent.”
2. Structural Abnormality Attribution Type	Attributing the problem to specific anatomical abnormalities in the upper airway.	P-C-05	2.1 Upper Airway Structural Issues (4)	“My sensation seems to originate from the nose—breathing congestion in the nasal passages. It basically starts with the nose becoming blocked.”
		P-C-01	2.2 Dominant Symptom Perception of Pharyngeal Collapse (2)	“During apnea episodes, it feels like something in my throat—like the back of my tongue collapses and blocks the airway, causing me to wake up.”
		P-C-03	2.3 Airway Obstruction Caused by Tongue Base Falling Back (2)	“I think the second scenario—deep collapse in the throat—closely matches my actual experience.”
3. Complex Comorbidity-Dominant Model	Positioning OSA within a network of severe systemic comorbidities, treating it as an integral part thereof.	P-C-07	3.1 Complex Comorbidity Cluster-Dominated OSA Narrative (5) 3.2 Cerebral infarction as a clear aggravating factor for OSA (2)	“Heart failure, cardiac insufficiency, cerebral infarction—these conditions lead to my sleep apnea.”
4. Physiological Event-Associated Type	Associating onset or exacerbation with specific physiological or life events.	P-C-04	4.1 Attribution Pointing to Hormonal Physiological Changes (1)	“Since this issue started after childbirth, I think the significant fluctuations in progesterone and estrogen levels might have played a small role.”
		P-C-08	4.2 Weight Change History as Core Initiating Factor (1) 4.3 Early-Onset Symptoms and Non-Obesity Period (1)	“There’s no direct link, really. It’s a long-term process. Drinking led to a beer belly, and only after gaining weight did this condition gradually develop.”
5. Other Dominant Types	Narratives are fragmented, with no clear dominant pattern emerging.	P-C-06	5.1 Integrated Perception of Pharyngitis and OSA (2)	(Regarding pharyngitis) They are one and the same thing; it’s a cycle.

Reference point counts derived from NVivo matrix coding queries; patient citations anonymized. Participant anonymization codes: P-C denotes Paradoxical Group participants (coded P-C-01 to P-C-08). Numbers in parentheses following key theme nodes indicate the number of coding reference points for that participant at that node. All 8 participants in this group had high BRI ( ≥ 5.6) and non-severe OSA (AHI < 30). Demographic and clinical characteristics are detailed in Supplementary Table S1.

#### 3.2.2 Intergroup comparison

Comparative analysis revealed systematic differences across four dimensions (Table 4):

**TABLE 4:** Comparative narrative themes between conflicted and typical groups.

Core categories	Contradiction group (*n* = 8)		Typical group (*n* = 17)	
	Subtheme (reference point)	Representative citation	Subtheme (reference point)	Representative citation
1. Attribution to BRI	Dispute of systemic respiratory burden mechanism (1)	P-C-01: “I don’t feel like my whole body is heavy and makes breathing difficult when I sleep—after all, I’m not that overweight.”	Fundamental Perception of Body Shape Impact (3)	P-T-03: “I feel a bit overweight. They say being overweight makes snoring more likely, and I think it’s related to my body type. Plus, my neck is a bit thick, which affects my throat and might be why my snoring is so loud.”
	Explicit Denial of Body Shape Influence (2)	P-C-06: “It’s not related to my body type. It’s more connected to issues like rhinitis and pharyngitis, plus my own irregular sleep schedule.”	Breathing Difficulty Due to Neck Compression (1)	P-T-04: “I’m overweight, and my neck is thick. That thickness presses down on my throat, making it hard for me to breathe.”
2. Core Symptom Experiences	Uncontrollable daytime sleepiness (1)	P-C-04: “I was mixing formula for my child, and while doing so, I started squinting my eyes and then just drifted off to sleep. When playing with him, I’d also fall asleep unconsciously in the middle of it.”	Daytime Functional Impairment (15)	P-T-07: “I fall asleep sitting down. As soon as I sit to rest, I fall asleep. During meetings, I’ve suddenly fallen asleep at least 10 times.”
	Acute neuromuscular suppression by alcohol (1)	P-C-08: “I snore constantly at night, but I don’t feel sleepy during the day. If I drink heavily, I do have trouble sleeping at night, and my breathing pauses become worse.”	Behavioral Rigidity and Cognitive Limitations (2)	P-T-05: “Since I usually do physical labor, I’ve grown accustomed to stronger flavors.”
3. Alternative Explanatory Model	OSA Narrative Dominated by Complex Comorbidity Clusters (5)	P-C-07: “Besides my body type, I also have hypertension, pharyngitis, heart failure, atrial fibrillation, cerebral infarction, and many other conditions.”	Atypical Body Type and Respiratory Mechanism Abnormalities (1)	P-T-05: “I have some limb deformities (from childhood paralysis), so I feel these deformities have a certain impact on my sleep breathing.”
	Integrated Understanding of Pharyngitis and OSA (1)	P-C-04:” I still think it’s the condition of the pharynx. Since I have chronic pharyngitis myself, it’s essentially the same issue—it’s a cycle.”	Structural Issues in the Upper Airway (25)	P-T-07: “If someone has chronic pharyngitis, my understanding is that it affects the respiratory tract. There are issues with nasal airflow and rhinitis, which can impact sleep.”
4. Behavior and Coping	Gender Differences in BRI and Behavioral Attribution (6)	P-C-03: “I think maybe more guys are overweight. I see them easily getting potbellies and snoring a lot.”	Positive Self-Education and Seeking Help (4)	P-T-03: “Drinking tea seems to help a bit. Don’t work too hard, lose weight, avoid rich foods—they make you gain weight. Don’t worry too much.”
	Cognitive-Behavioral Gap and Stress Barriers (1)	P-C-08: “Try to cut down on smoking and drinking as much as possible, but I still smoke when the pressure gets too high.”	Self-Management Behavioral Strategies (4)	P-T-12: “Persist with sleep monitoring, weight loss, and rest as much as possible.”

Contradictory Group (*n* = 8) is defined as patients with high visceral adiposity (BRI ≥ 5.6) and non-severe OSA (AHI < 30 events/h). Typical Group (*n* = 17) is defined as patients with high visceral adiposity (BRI ≥ 5.6) and severe OSA (AHI ≥ 30events/h). Numbers in parentheses indicate NVivo coding reference points. P-C denotes Contradictory Group participants (P-C-01 to P-C-08); P-T denotes Typical Group participants (P-T-01 to P-T-17).

Attribution of BRI: The Typical Group accepted central obesity as the fundamental cause, whereas the Contradictory Group explicitly denied this association (e.g., “It’s not related to my body shape; it’s more related to rhinitis and irregular schedule” [P-C-06]).

Symptom experience: The Typical Group reported uncontrollable daytime sleepiness and functional impairment (e.g., “I fall asleep sitting down. I’ve dozed off during meetings at least ten times” [P-T-07]). The Contradictory Group reported non-specific nocturnal discomfort without daytime fatigue (e.g., “I snore at night, but I’m not sleepy during the day” [P-C-08]).

Behavioral Coping: The Typical Group favored active management (e.g., weight loss, monitoring), while the Contradictory Group exhibited passive adaptation and cognitive-behavioral gaps (e.g., “Trying to cut down on smoking, but still smoke when stressed” [P-C-08]).

Gendered Cognition: Female participants frequently dismissed body shape impacts by citing sex differences in fat distribution (e.g., “Guys gain weight more easily—I see them getting potbellies and snoring” [P-C-03]), providing a sociocultural explanation for the lower diagnostic efficacy of BRI in women observed quantitatively.

### 3.3 Mixed-methods integration

**FIGURE 8:**
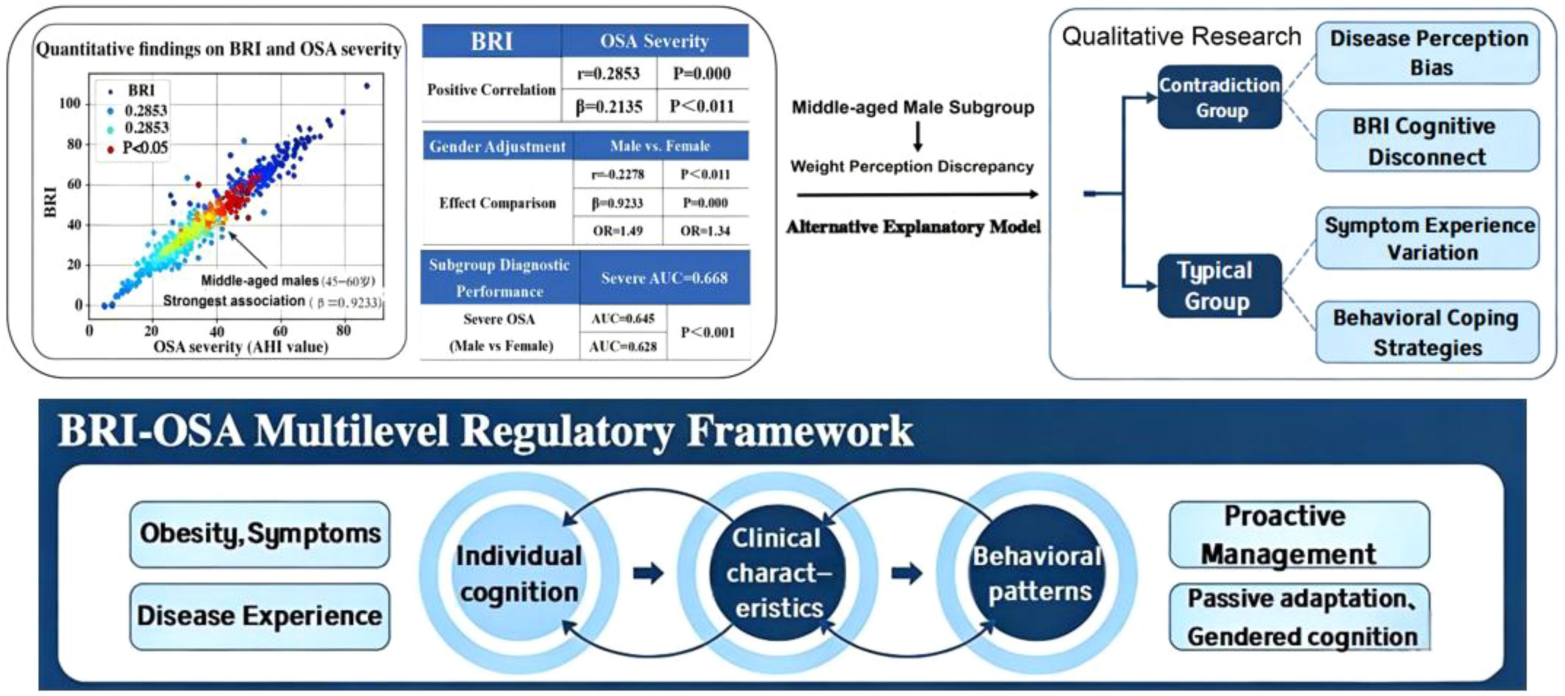
Data integration visualization. The joint display juxtaposes quantitative results (left panel: statistical association, subgroup disparities) with qualitative insights (right panel: disease perception bias, cognitive disconnect, and coping patterns) to resolve the “high BRI without severe OSA” paradox. The bottom panel represents the derived BRI-OSA Multilevel Regulatory Framework integrating individual cognition, clinical characteristics, and behavioral outcomes.

A visual joint display synthesized quantitative and qualitative findings (Figure 8). Quantitative moderation effects (sex, age, comorbidities) were cross-validated by qualitative themes (alternative explanatory models, gendered cognition). The Contradictory Group’s structured alternative attributions directly elucidated why BRI’s diagnostic efficacy weakens in mild-to-moderate OSA and specific comorbid populations. Specifically, the quantitative phase identified a paradoxical subgroup with high BRI yet non-severe OSA. Qualitative inquiry revealed that these individuals systematically attributed symptoms to localized upper airway issues (e.g., chronic pharyngitis, nasal congestion) or comorbidities rather than systemic obesity. This divergence in disease attribution directly informed the proposed screening pathway by justifying the incorporation of a structured assessment of individual health beliefs and upper airway comorbidities as an explicit step, thereby transforming the pathway from a purely metric-driven algorithm into a patient-centered, multidimensional framework. This integration establishes a multilevel regulatory framework demonstrating that BRI functions not as a universal screening tool, but as one whose efficacy is heavily dependent on individual disease cognition, clinical characteristics, and behavioral patterns.

## 4 Discussion

### 4.1 Diagnostic efficacy boundaries of BRI

This explanatory sequential mixed-methods study delineates the diagnostic boundaries of the Body Roundness Index (BRI) for OSA screening. In severe OSA, BRI demonstrated comparable efficacy to BMI (AUC = 0.668 vs. 0.674), but both were significantly outperformed by neck circumference (AUC = 0.711). This convergence likely stems from high collinearity between systemic and central obesity in severe phenotypes, where mechanical obstruction dominates (30–32). Notably, BRI showed limited discriminatory ability for mild-to-moderate OSA (AUC < 0.55), supporting the consensus that screening for less severe phenotypes must integrate broader clinical contexts beyond singular morphological metrics (32). Our observation that neck circumference outperformed systemic body indices such as BMI and BRI in predicting severe OSA aligns with recent findings by Topaloglu et al., who demonstrated that localized, upper-body adipose deposition remains a more direct anatomical driver of upper airway collapse than general roundness or BMI (33).

We must emphasize that BRI should be regarded as a complementary anthropometric indicator rather than a superior screening tool. While neck circumference captures direct upper airway constriction, BRI uniquely reflects abdominal visceral adiposity and general metabolic burden. The combined model (BRI + BMI + neck circumference) yielded only a modest AUC (0.689) that did not surpass neck circumference alone (0.711). From a clinical efficiency perspective, calculating multiple collinear obesity indices offers redundant information. Thus, BRI’s primary utility may lie in providing a pathophysiologically interpretable alternative to BMI when waist circumference data are available, particularly for assessing systemic cardiometabolic risk alongside OSA screening, rather than enhancing standalone diagnostic accuracy.

### 4.2 Gender disparities: intertwined physiological and psychosocial mechanisms

A standout finding is the gender disparity in BRI’s diagnostic efficacy (males: AUC = 0.645; females: AUC = 0.628), driven by dual dimensions. Physiologically, men’s visceral, upper-body fat accumulation directly correlates with airway compression, whereas premenopausal women’s peripheral fat distribution weakens this association (34, 35). Qualitatively, female patients frequently attributed symptoms to hormonal fluctuations or nonspecific causes, actively rejecting the “obesity-snoring” paradigm. This cognitive disparity delays consultation and biases reporting, diluting the statistical association between obesity indicators and disease severity—aligning with prior evidence of concealed OSA symptoms and underdiagnosis among women (36).

### 4.3 Toward patient-centered, multidimensional screening

Crucially, combining BRI, BMI, and neck circumference did not surpass neck circumference alone (AUC: 0.689 vs. 0.711), confirming information redundancy among collinear indicators (37, 38). The absence of meaningful incremental discriminative improvement suggests that aggregating multiple obesity-related metrics is unlikely to yield clinically significant gains in routine screening practice. Instead, the potential added value of BRI may lie in its pathophysiological interpretability as a continuous marker of central adiposity when neck circumference measurement is impractical, rather than in enhancing diagnostic accuracy beyond existing tools. Qualitative findings further revealed that non-obesity factors—pharyngitis history, nasal symptoms, alcohol consumption—frequently drive symptom attribution in the “Contradictory Group.” These patient-narrated themes directly operationalized the proposed screening pathway: by identifying that individuals with high BRI but non-severe OSA systematically attribute symptoms to localized upper airway issues or comorbidities rather than systemic obesity, the pathway explicitly incorporates structured inquiry into upper airway comorbidities, lifestyle habits, and patients’ primary health concerns as engagement anchors. This exemplifies how qualitative insights were translated into actionable clinical components, supporting a shift from “indicator aggregation” to “heterogeneous risk integration” (39).

We propose a stepwise, patient-centered pathway: (1) Initial screening: Prioritize neck circumference; use BRI/BMI as alternatives when impractical; (2) Risk assessment: For positive or symptomatic patients, integrate structured interviews covering symptom profiles, upper airway comorbidities, lifestyle habits, and health concerns; (3) Clinical decision-making: Leverage the patient’s primary health concern as an engagement anchor to enhance adherence. This transforms quantitative indicators into components of a responsive clinical narrative.

### 4.4 Strengths, limitations, and future directions

The core strength lies in the mixed-methods design, which transcends statistical validation to explore why and how indicators perform in specific contexts (40). However, several limitations should be acknowledged. First, the cross-sectional design precludes causal inference and temporality assessment between BRI and OSA severity. Second, the single-center sample, drawn from a tertiary sleep medicine center in southern China, and the relatively small female proportion (23.4%) may limit generalizability to broader primary care populations or to settings with different ethnic and sex distributions. Third, although BRI provides a continuous measure of central obesity, its calculation depends on standardized waist circumference measurement, which may be susceptible to inter-operator variability in routine clinical practice. Fourth, the qualitative phase relied on purposive sampling of patients with high BRI; perspectives of individuals with low BRI but severe OSA were not captured, potentially introducing selection bias. Fifth, while information saturation was declared after 25 interviews, the small subgroup sizes (*n* = 8 and *n* = 17) may have limited the detection of rare narrative themes. Sixth, we acknowledge that multicollinearity diagnostics, though reported, reflect *post-hoc* verification; future studies should pre-specify collinearity thresholds when simultaneously modeling mathematically related anthropometric indices. Seventh, several potential confounders—including detailed physical activity levels, dietary habits, and genetic predisposition—were not systematically recorded and could independently influence both body morphology and respiratory parameters. Finally, the proposed patient-centered screening pathway remains theoretical and requires prospective validation through implementation studies to assess its impact on detection rates, referral efficiency, and patient adherence. Future multicenter prospective cohorts should validate BRI’s predictive value, and randomized trials are needed to evaluate the proposed pathway’s impact on detection rates and referral efficiency.

## 5 Conclusion

While BRI offers a pathophysiologically grounded alternative to BMI for severe OSA risk assessment, it does not surpass neck circumference. Its clinical utility is highly context-dependent, modulated by physiological gender differences and psychosocial factors—particularly patient disease attribution models. Simply aggregating collinear obesity indicators is inefficient. Instead, we advocate for a multidimensional, patient-centered screening paradigm that prioritizes neck circumference while systematically incorporating individual clinical characteristics and health beliefs to achieve precise, actionable OSA risk assessment.

## Data Availability

The raw data supporting the conclusions of this article will be made available by the authors, without undue reservation.
