# Supplementary material for: The impact of body roundness index (BRI) on obstructive sleep apnea (OSA) severity: an exploratory sequential mixed-methods study
Source: Front Med (Lausanne). 2026 Jul 29;13:1885036. doi: 10.3389/fmed.2026.1885036 (PMC13461924; doi:10.3389/fmed.2026.1885036)
Supplement: Supplementary file 1 [file Supplementary_file_1.docx]

**Supplementary Materials**

**S1. Detailed Sample Size Estimation**

This study is a cross-sectional observational study. The sample size estimation was based on the standard formula for estimating a population prevalence^[41]^:

$$n=\frac{\left[ Z_{\alpha/2}\sqrt{p(1-p)}+Z_{\beta}\sqrt{p(1-p)} \right]^{2}}{d^{2}}$$

Where:

Z is the statistical value corresponding to the confidence level (set at 1.96 for a 95% confidence interval, two-tailed test with α=0.05 )

P is the expected prevalence. Based on relevant epidemiological literature, the prevalence of obstructive sleep apnea (OSA) ranges from 9% to 38% and shows an increasing trend annually. Therefore, we assumed a conservative prevalence rate (p ) of 38% (0.38) to ensure adequate statistical power

d is the acceptable margin of error, set at 0.04 to ensure high precision

Substituting these parameters into the formula yields:

$$n=\frac{{1.96}^{2}\times0.38\times0.62}{{0.04}^{2}}\approx566$$

Accounting for potential invalid or missing data, the sample size was expanded by approximately 10%, resulting in a minimum required sample size of 622 cases. Ultimately, this study successfully enrolled 2,596 participants, far exceeding the requirement and ensuring ample statistical power for subsequent multivariate regression and subgroup analyses.

**S2. Calculation of the Body Roundness Index (BRI)**

The core exposure variable, Body Roundness Index (BRI), was calculated using the following established formula:

$$BRI=364.2-365.5\times\sqrt{1-\frac{\left( {WC}/{2\pi} \right)^{2}}{\left( 0.5\times H \right)^{2}}}$$

Where:

WC represents waist circumference, measured in centimeters (cm) and converted to meters (m) for calculation

H represents height, measured in centimeters (cm) and converted to meters (m) for calculation

Height and waist circumference were measured using standard soft tape measures, accurate to 0.1 cm.

**S3. Semi-Structured Interview Guide (Outline)**

The qualitative phase utilized a semi-structured interview guide developed based on the Phase I quantitative findings. The core questions explored included:

Disease Attribution: "What do you believe is the main cause of your snoring or breathing pauses during sleep?"

Symptom Experience: "Can you describe how your sleep issues affect your daytime functioning or overall well-being?"

Perceptions of Body Shape (BRI): "Some studies suggest that body shape or weight is related to sleep apnea. Do you feel your body shape has impacted your sleep? Why or why not?"

Behavioral Coping Strategies: "What actions, if any, have you taken to manage your sleep issues or weight?"

**S4. Detailed Scoring Criteria for OSA Assessment Tools**

Epworth Sleepiness Scale (ESS): Total score 0–24; 0-9 = normal, 10-12 = mild, 13-15 = moderate, ≥16 = severe daytime sleepiness.

Berlin Questionnaire: Comprises three dimensions (snoring, daytime sleepiness, hypertension/obesity); high risk in ≥2 dimensions indicates high OSA risk.

**Figure S1: Heatmap of Correlations Between Continuous Variables and OSA Severity**

Figure S1: Heatmap of pairwise correlations among continuous clinical and anthropometric variables.

Note: The correlation heatmap illustrates the Pearson/Spearman correlation coefficients (r) among continuous clinical parameters and anthropometric indices of the study population. The color bar on the right defines the correlation strength and direction: shades of blue represent positive correlations (up to 1.0), shades of red/orange represent negative correlations (down to -1.0), and white indicates the absence of a linear correlation (close to 0.0).

**Table S1: Predictive Performance of BRI, BMI, and Neck Circumference for Different Severity Levels of OSA**

| Prediction  Indicator | Subgroup | OSA  Severity | AUC (95% CI) | Optimal Cutoff | Sensitivity (%) | Specificity (%) | P-value | Remarks |
| --- | --- | --- | --- | --- | --- | --- | --- | --- |
| **Body Roundness**  **Index (BRI)** |  | Mild (5 ≤ AHI < 15) | 0.532 (0.526–0.620) | 5.60 | 62.7 | 50.2 | 0.0015 | Control: Normal (AHI < 5) |
|  |  | Moderate (15 ≤ AHI < 30) | 0.490 (0.426–0.570) | 2.32 | 60.4 | 52.2 |  |  |
|  |  | **Severe (AHI ≥ 30)** | **0.668 (0.642–0.722)** | **4.75** | **62.8** | **68.5** |  |  |
|  | **Male** | **Severe (AHI ≥ 30)** | **0.645 (0.621–0.670)** | **4.55** | **60.4** | **60.8** | <0.001 |  |
|  | **Female** | **Severe (AHI ≥ 30)** | **0.628 (0.579–0.677)** | **2.50** | **60.8** | **60.4** |  |  |
| **Body Mass Index (BMI)** |  | Mild (5 ≤ AHI < 15) | **0.458 (0.434–0.482)** | 3.57 | 57.6 | 59.8 | 0.0037 | Control: Normal (AHI < 5) |
|  |  | Moderate (15 ≤ AHI < 30) | **0.474 (0.447–0.501)** | 4.21 | 70.9 | 65.8 |  |  |
|  |  | Severe (AHI ≥ 30) | **0.674 (0.653–0.695)** | 2.50 | 47.7 | 79.2 |  |  |
|  | **Male** | **Severe (AHI ≥ 30)** | **0.632 (0.609–0.655)** | 2.50 | 39.9 | 80.3 | 0.012 |  |
|  | **Female** | **Severe (AHI ≥ 30)** | **0.594 (0.547–0.642)** | 2.50 | 35.5 | 81.8 |  |  |
| **Neck Circumference** |  | Mild (5 ≤ AHI < 15) | **0.447 (0.421–0.472)** | 29.50 | 98.3 | 2.2 | 0.006 | Control: Normal (AHI < 5) |
|  |  | Moderate (15 ≤ AHI < 30) | **0.507 (0.480–0.545)** | 37.25 | 61.2 | 43.3 |  |  |
|  |  | Severe (AHI ≥ 30) | **0.711 (0.690–0.733)** | 39.50 | 59.7 | 72.6 |  |  |
| **BRI + BMI + Neck Circumference** |  | Severe (AHI ≥ 30) | **0.689 (0.669–0.709)** | **0.48** | **68.4** | 60.8 | <0.001 | Good (H-L test P=0.342) |

Note: Control group was defined as Normal (AHI< 5 events/h).P-values indicate the significance of diagnostic indicators. H-L test refers to the Hosmer-Lemeshow goodness-of-fit test, where P>0.05 indicates an acceptable model fit.

****Table S2: Qualitative Interview Participant Information Table****

| ID | Research Group | Gender | Age (years) | BRI | OSA | Core Narrative Type  (Conflict Group) | Dominant Narrative Category  (Representative of the Archetypal Group) |
| --- | --- | --- | --- | --- | --- | --- | --- |
| P-C-01 | Contradiction Group | Male | 40 | 5.8 | Moderate | Structural Abnormality Attribution Type | — |
| P-C-02 | Contradiction Group | Female | 50 | 6.4 | Moderate | Local Symptom Attribution Type | — |
| P-C-03 | Contradiction Group | Female | 38 | 7.5 | Mild | Structural Abnormality Attribution Type | — |
| P-C-04 | Contradiction Group | Female | 35 | 9.7 | Moderate | Physiological Event-Associated Type | — |
| P-C-05 | Contradiction Group | Female | 30 | 7.4 | Moderate | Structural Abnormality Attribution Type | — |
| P-C-06 | Contradiction Group | Female | 48 | 6.6 | Mild | Other (pharyngitis predominant) | — |
| P-C-07 | Contradiction Group | Female | 69 | 7.8 | Mild | Complex Comorbidity Dominant Type | — |
| P-C-08 | Contradiction Group | Male | 42 | 8.9 | Moderate | Physiological Event-Associated Type | — |
| **P-T-01** | **Typical group** | **Male** | **35** | **8.5** | **Severe** | **—** | **Attribution to BRI** |
| P-T-02 | Typical Group | Male | 40 | 6.8 | Severe | — | — |
| P-T-03 | Typical Group | Male | 58 | 7.5 | Severe | — | Attribution to BRI |
| P-T-04 | Typical group | Male | 61 | 10.4 | Severe | — | Core Symptom Experience |
| P-T-05 | Typical Group | Male | 32 | 9.8 | Severe | — | Alternative Explanation Model |
| P-T-06 | Typical Group | Male | 36 | 6.9 | Severe | — | Attribution to BRI |
| P-T-07 | Typical Group | Male | 45 | 6.7 | Severe | — | Alternative Explanation Model |
| P-T-08 | Typical Group | Male | 29 | 7.2 | Severe | — | Core Symptom Experience |
| P-T-09 | Typical Group | Male | 40 | 8.6 | Severe | — | — |
| P-T-10 | Typical group | Male | 56 | 7.1 | Severe | — | — |
| P-T-11 | Typical Group | Female | 51 | 6.8 | Severe | — | — |
| P-T-12 | Typical group | Female | 38 | 7.7 | Severe | — | Behavior and Coping |
| P-T-13 | Typical Group | Female | 32 | 9.3 | Severe | — | — |
| P-T-14 | Typical group | Female | 61 | 7.4 | Severe | — | — |
| P-T-15 | Typical group | Female | 49 | 6.3 | Severe | — | — |
| P-T-16 | Typical group | Female | 30 | 7.3 | Severe | — | — |
| P-T-17 | Typical group | Female | 54 | 9.8 | Severe | — | — |

Note: P-C denotes participants in the Contradictory Group (high visceral adiposity with non-severe OSA). P-T denotes participants in the Typical Group (high visceral adiposity with severe OSA). Horizontal lines (—) indicate that the corresponding column category or narrative type is not applicable to that specific participant.

**References**

41.	Whelton, P. K., & Gordis, L. (2000). Epidemiology of clinical medicine. *Epidemiologic Reviews*, *22*(1), 140–144. https://doi.org/10.1093/oxfordjournals.epirev.a018012
